# A Randomised, Controlled Study of Different Glycaemic Targets during Gestational Diabetes Treatment: Effect on the Level of Adipokines in Cord Blood and ANGPTL4 Expression in Human Umbilical Vein Endothelial Cells

**DOI:** 10.1155/2018/6481658

**Published:** 2018-05-14

**Authors:** P. Popova, L. Vasilyeva, A. Tkachuck, M. Puzanov, A. Golovkin, Y. Bolotko, E. Pustozerov, E. Vasilyeva, O. Li, I. Zazerskaya, R. Dmitrieva, A. Kostareva, E. Grineva

**Affiliations:** ^1^Almazov National Medical Research Centre, Saint Petersburg, Russia; ^2^Department of Internal Diseases and Endocrinology, St. Petersburg Pavlov State Medical University, Saint Petersburg, Russia; ^3^Department of Biomedical Engineering, Saint Petersburg State Electrotechnical University, Saint Petersburg, Russia

## Abstract

Our aim was to study the expression of adipokine-encoding genes (leptin, adiponectin, and angiopoietin-like protein 4 (ANGPTL4)) in human umbilical vein endothelial cells (HUVECs) and adipokine concentration in cord blood from women with gestational diabetes mellitus (GDM) depending on glycaemic targets. GDM patients were randomised to 2 groups per target glycaemic levels: GDM1 (tight glycaemic targets, fasting blood glucose < 5.1 mmol/L and <7.0 mmol/L postprandial, *N* = 20) and GDM2 (less tight glycaemic targets, <5.3 mmol/L and < 7.8 mmol/L, respectively, *N* = 21). The control group included 25 women with normal glucose tolerance. ANGPTL4 expression was decreased in the HUVECs from GDM patients versus the control group (23.11 ± 5.71, 21.47 ± 5.64, and 98.33 ± 20.92, for GDM1, GDM2, and controls; *p* < 0.001) with no difference between GDM1 and GDM2. The level of adiponectin gene expression was low and did not differ among the groups. Leptin gene expression was undetectable in HUVECs. In cord blood, leptin/adiponectin ratio (LAR) was increased in GDM2 compared to controls and GDM1 (*p* = 0.038) and did not differ between GDM1 and controls. Tight glycaemic targets were associated with normalisation of increased LAR in the cord blood. ANGPTL4 expression was downregulated in HUVECs of newborns from GDM mothers and was not affected by the intensity of glycaemic control.

## 1. Introduction

The intrauterine hyperglycaemia in women with gestational diabetes mellitus (GDM) is supposed to be an important factor that predisposes offspring to obesity and type 2 diabetes mellitus (T2D) [1, 2]. However, the mechanisms connecting intrauterine exposure to hyperglycaemia with subsequent development of metabolic diseases are not clear enough.

There is some suggestion that the exposure to diabetes in utero increases the risk of offspring obesity via alterations in the “adipoinsular axis,” the endocrine loop, linking the brain and endocrine pancreas with insulin- and leptin-sensitive tissues in the control of eating behaviour and energy balance [1, 3].

Adipokines play an important role in the energy metabolism regulation [4]. Leptin (LEP) and adiponectin (ADIPOQ) are well-recognised obesity- and diabetes-related candidate genes through which the adipose tissue influences the regulation of several important physiological functions, including appetite, satiety, energy expenditure, insulin sensitivity, fat distribution, and endothelial function [4]. Adiponectin and leptin are also factors associated with fetal growth [5] and shown as predictors of early-life weight gain [6, 7].

Another promising adipokine is angiopoietin-like protein 4 (ANGPTL4), a multifunctional signal protein expressed in many tissues. ANGPTL4 is involved in the regulation of multiple physiological processes, including energy metabolism [8], plasma glucose level and tolerance regulation [9], fat storage, and lipid metabolism [10]. The association of ANGPTL4 expression with obesity was confirmed in a study of monozygotic twins [11]. Robciuc et al. revealed that ANGPTL4 expression in the adipose tissue and circulation was inversely correlated to body weight, suggesting a role for ANGPTL4 in acquired obesity [11].

The change in the expression of the abovementioned genes in the fetal tissues may serve as a marker of subsequent metabolic diseases of the offspring. The association between the presence of hyperglycaemia in the mother and altered cord blood levels of leptin, adiponectin, and ANGPTL4 has been identified in previous studies [5, 12, 13]. An increased placental LEP expression level has been also described in women with GDM [14].

However, it is not obvious that maternal hyperglycaemia causes such alterations. Perhaps, on the contrary, the altered gene expression functions in GDM pathogenesis (e.g., due to activation of hormone-encoding genes evoking insulin resistance or reduction of insulin secretion). It is also possible that both phenomena (maternal hyperglycaemia and changes in the expression of adipokines in the fetus and/or the mother) result from other pathological processes.

Randomised controlled trials (RCT) comparing changes of newborn gene expression level in groups of women with different target glucose levels during the treatment of GDM are supposed to help clarifying the cause-and-effect relations.

The human umbilical vein endothelial cells (HUVECs) represent a good cellular model for studying the effect of maternal hyperglycaemia on the fetal cardiovascular system and can serve as a marker of the predisposition of the fetus to metabolic diseases [15].

In this study, we investigated the alterations in ANGPT4, ADIPOQ, LEP, and leptin receptor gene (LEPR) expression levels in HUVECs and concentrations of these adipokines in the cord blood from newborns of women with GDM with different glycaemic targets compared to healthy women.

## 2. Materials and Methods

This study was carried out at the Almazov National Medical Research Centre (ANMRC) as part of the ongoing RCT “Genetic and epigenetic mechanisms of developing gestational diabetes mellitus and its effects on the fetus” (GEM GDM) which started in July 2015. This study was approved by the local ethical committee (Protocol 119); informed written consent was obtained from all subjects.

### 2.1. Design and Study Population

Forty-one women with GDM and 25 controls were randomly selected to assess the levels of expression of genes in HUVECs. The women with GDM were randomised to 2 groups according to target glycaemic levels: group 1 (target fasting blood glucose < 5.1 mmol/L and <7.0 mmol/L 1-hour postprandial) (GDM1, *N* = 21) and group 2 (target fasting blood glucose < 5.3 mmol/L and <7.8 mmol/L 1-hour postprandial) (GDM2, *N* = 20).

GDM was diagnosed according to the Russian National Consensus [16] and the recommendations of the International Association of Diabetes and Pregnancy Study Groups (IADPSG) based on the results of 2-hour oral glucose tolerance test (OGTT) performed at 24th–28th week of gestation [17]. Pregnant women without diabetes were included as controls.

None of the patients had previous history of diabetes mellitus or any known medical condition affecting glucose metabolism.

They were all followed until delivery at ANMRC. Anthropometric variables (height and blood pressure) were measured using standardised procedures. Prepregnancy body mass index (BMI) was calculated based on the prepregnancy weight recalled by participants. Women with GDM were consulted by the endocrinologist and provided the results of their self-measurements of blood glucose every 2-3 weeks. In case of exceeding the target blood glucose levels (in 2 or more measurements per week in group 1 and in more than 1/3 of measurements per week in group 2), insulin therapy was started. The participants were asked to keep electronic nutrition and glycaemic control diaries with the help of a specially developed mobile application and send data to the doctor. The mobile application is described elsewhere [18]. According to the personal diaries, automatic calculations of the integral indicators characterising the self-control of glycaemia (fasting, postprandial, and average glycaemia) were accomplished. Electronic diary data were available for almost all women with GDM (*N* GDM1 = 19, *N* GDM2 = 20) and 8 women from the control group.

### 2.2. Blood Sample Processing and Analysis

Cord blood samples were collected immediately after delivery. Blood glucose measurements were made on fresh plasma samples. The cord blood serum samples were stored at −80°C for further analysis of C-peptide, leptin, adiponectin, and ANGPTL4. Plasma glucose (PG) concentration was determined by the glucose oxidase method. Serum C-peptide level was measured by the chemiluminescent microparticle immunoassay (Architect C-peptide assay, Abbott Laboratories, IL, USA). Serum adiponectin (BioVendor Laboratory Medicine Inc., Modrice, Czech Republic) and leptin (Diagnostics Biochem Canada Inc., Canada) levels were measured using an enzyme-linked immunosorbent assay (ELISA) as recommended by the manufacturer. Serum level of ANGPTL4 was determined by DuoSet ELISA Development kits (DY3485) from R&D Systems (USA). The limit of detection for ANGPTL4 is 1.25 ng/mL. The detection range is 1.25 ng/mL–80 ng/mL. The following factors prepared at 800 ng/mL were assayed and exhibited no cross-reactivity or interference: recombinant human angiopoietin-1, angiopoietin-2, angiopoietin-4, and angiopoietin-like 3 and recombinant mouse angiopoietin-3 and angiopoietin-like 3.

The limit of detection for Leptin is 0.5 ng/mL. The detection range is 0.5–100 ng/mL.

The following substances were tested at 1000 ng/mL and exhibited no cross-reactivity: mouse leptin, TNF-α, IL-2, IL-3, IL-4, IL-6, IL-8, IL-9, IL-10, IL-12, IL-16, GM-CSF, CSF, and EGF.

The limit of detection for adiponectin is 26 ng/mL. The detection range is 26 ng/mL–100 ug/mL. No cross-reactivity has been observed for human leptin and leptin receptor.

Intra-assay coefficients of variation (CVs) for leptin assay were between 3.7% and 5.5%, and interassay CVs were 5.8–6.8%. For adiponectin assay, the intra- and interassay CVs were 3.9–5.9% and 6.3–7.0%, respectively.

### 2.3. Isolation and Identification of the HUVECs

The HUVECs were isolated using a standard collagenase digestion method [19] as we do routinely in our laboratory [20]. Immediately after isolation, the cells were cultured and expanded in endothelial cell medium (ECM cat number 1001, ScienCell, San Diego, CA) containing 5% fetal bovine serum, 1% penicillin/streptomycin, and endothelial cell growth supplement in a humidified atmosphere of 95% air and 5% CO_2_ at 37°C. For this study, 80% of confluent HUVEC monolayers (passages 2-3) were used.

The purity of primary HUVEC cultures was evaluated by flow cytometry analysis performed on Guava EasyCyte8. Briefly, detached cells were resuspended in 200 *μ*L of PBS containing 1% bovine serum albumin (Sigma-Aldrich, Saint Luis, MO, USA) and incubated for 15 min at 20°C with the following antibodies (Ab): FITC-conjugated anti-CD31, PE-A-conjugated anti-CD144, PE-Cy7-A-conjugated anti-CD146 (BioLegend, San Diego, CA, USA), PE-A-conjugated anti-CD105 (Bioscience Pharmingen, San Jose, CA, USA), and APC-A-conjugated anti-CD45 (DAKO, Santa Clara, CA, USA). Data files were collected and analysed using the FACSDiva software program (version 6.1.3; BD Bioscience, San Jose, CA, USA).

### 2.4. Evaluation of Apoptosis and Immunocytochemical Assay

The viability of HUVEC was assessed by flow cytometry with the determination of the number of viable cells, as well as those in early and late apoptosis and necrosis evaluated by Annexin-V/PI (BioLegend, San Diego, CA, USA) double staining.

The expression of von Willebrand factor and CD146 (BioLegend, San Diego, CA, USA) in HUVECs was detected by immunocytochemical staining. Cell nucleuses were stained with 4′,6-diamidino-2-phenylindole (DAPI).

### 2.5. RT-qPCR

Total RNA was extracted from HUVEC using ExtractRNA reagent (BC032, Evrogen, Moscow, Russia) according to the manufacturer's protocol. One microgram of total RNA was reverse transcribed using Moloney Murine Leukemia Virus Reverse Transcriptase (MMLV RT) kit (SK021, Evrogen, Moscow, Russia). After cDNA synthesis, quantitative real-time PCR was performed in 25 *μ*L reaction mixture containing: 5x qPCRmix-HS LowROX (PK154L, Evrogen, Moscow, Russia) diluted to a final concentration of 1x, 20x primers diluted to 1x, 50 ng cDNA, and deionised distilled water. Reaction mixtures were incubated for an initial denaturation at 95°C for 10 min, which was followed by 40 PCR cycles, each consisting of exposure to 95°C for 15 sec and 60°C for 1 min. Gene expression was evaluated by real-time PCR using Applied Biosystems TaqMan Gene Expression Assays (ADIPOQ: Hs00605917_m1; LEP: Hs00174877_m1; LEPR: Hs00174497_m1; and ANGPTL4: Hs01101127_m1). All data are expressed as ratio to the reference gene GAPDH (forward AATGAAGGGGTCATTGATGG, reverse AAGGTGAAGGTCGGAGTCAA) (AlkorBio, Saint-Petersburg, Russia).

Relative expression was evaluated according to the 2^−ΔΔCt^ method [21]. In order to confirm the correctness of the method of detection of LEP and ADIPOQ expression, we used RNA samples derived from our previous adipose differentiation experiments [22]. RNA samples from adipose tissue multipotent mesenchymal stromal cells (MSC) and from differentiated in vitro adipose tissue were used as negative and positive controls, respectively.

### 2.6. Data Analysis

Statistical analysis was performed using SPSS 22.0 (SPSS Inc., USA). Mean and standard deviation are reported for continuous variables, and numbers and percentages are reported for categorical variables. Differences among the groups were analysed by Mann–Whitney test (for comparison between two groups), Kruskal-Wallis test (for comparison of more than two groups) or chi-square test. A *p* value < 0.05 was considered statistically significant.

## 3. Results

### 3.1. Characteristics of the Participants

Baseline characteristics of the participants are described in Table 1. The women from all three groups did not differ in terms of age and prepregnancy BMI. The GDM1 group had higher diastolic BP compared to controls (*p* = 0.003). The GDM1 and GDM2 groups had higher levels of fasting PG (*p* = 0.004 and *p* = 0.003, resp.) and higher levels of PG 1 h and 2 h in OGTT (*p* < 0.001 in comparison with controls).

Mean levels of fasting, 1-hour postprandial and average blood glucose measured by the participants during the study are described in Table 2. The GDM1 group achieved significantly lower average and 1-hour postprandial glucose levels compared to GDM2. Mean postprandial BG was lower in GDM1 even compared to the control group, though the difference did not reach statistical significance (*p* = 0.088). Gestational weight gain did not differ between GDM1 and GDM2 groups and was lower in both groups compared to controls (Table 2). The percentage of women treated with insulin was 40% and 29% in the GDM1 and GDM2 groups, respectively, and did not significantly differ (*p* = 0.495).

### 3.2. Pregnancy Outcomes

Pregnancy outcomes and biochemical markers in cord blood are shown in Table 3.

There was no statistically significant difference among the groups in terms of pregnancy outcomes (percent of large for gestational age (LGA) and small for gestational age (SGA) newborns, delivery by caesarean section) and the level of C-peptide, adiponectin, and ANGPTL4 in cord blood serum and glucose in cord blood plasma. The level of leptin in cord blood serum was higher in the GDM2 group than in the GDM1 (*p* = 0.036) and the control group, but the difference from the control group did not reach statistical significance (*p* = 0.066). After adjustment by insulin therapy, age, and prepregnancy BMI, the difference in the level of leptin between GDM1 and GDM2 remained significant (*p* = 0.01). The leptin/adiponectin ratio (LAR) in cord blood serum was higher in the GDM2 group compared to controls (*p* = 0.011) with no difference between the GDM1 and control group (*p* = 0.404).

### 3.3. HUVEC Characterisation

HUVECs were obtained from the umbilical vein, expanded in vitro, and characterised for expression of endothelial markers by flow cytometry and immunohistochemistry. All samples demonstrated characteristic endothelial morphology and immunophenotype CD45−/CD144+/CD31+/CD146+/CD105+ and stained positively for endothelial markers, von Willebrand factor, and CD146 (as demonstrated in our previous work [20]). There was no difference in the parameters of viability and replicative aging of HUVEC cultures from different patient groups (data not presented).

### 3.4. Gene Expression in HUVECs

**Figure 1:**
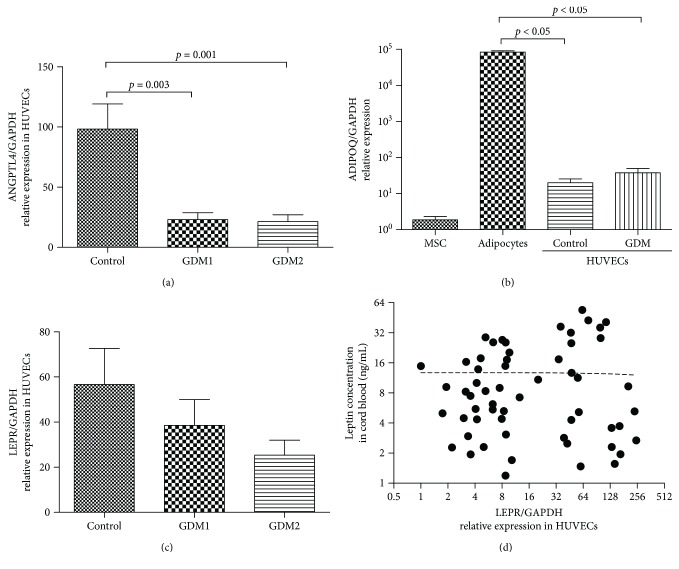
Gene expression analysis. (a) The level of relative ANGPTL4 mRNA expression in HUVECs from healthy (control) and GDM patients. (b) The level of relative ADIPOQ mRNA expression in multipotent mesenchymal stromal cells (MSC) (as negative control), adipocytes (as positive control), and HUVECs. (c) The level of relative LEPR mRNA expression in HUVECs from healthy (control) and GDM patients. (d) Correlation between the relative LEPR mRNA expression in HUVECs and the level of leptin in the cord blood.

ANGPTL4 expression was downregulated in the HUVECs derived from GDM patients compared to control group (23.11 ± 5.71, 21.47 ± 5.64, and 98.33 ± 0.92, respectively, for GDM1, GDM2, and control groups; *p* < 0.001 for comparison among the 3 groups) while no difference between GDM1 and GDM2 groups was observed (Figure 1(a)).

We did not detect the expression of LEP in HUVEC but found out that they expressed LEPR, and the expression of LEPR demonstrated a decline in the GDM1/GDM2 groups compared to the control group, though the differences were not statistically significant (Figure 1(c)). The expression of LEPR did not correlate with the level of LEP in cord plasma (Figure 1(d)) which indicates that there is no reciprocal regulation between LEP and its receptor in HUVECs. The expression of ADIPOQ was detected in HUVEC samples, but the level of its expression was as low as in negative control samples and lower by four orders of magnitude in HUVEC compared to adipocytes (positive control) (Figure 1(b)).

## 4. Discussion

Our RCT has shown that GDM treated according to the most widely accepted current guidelines was associated with the increased LAR in cord blood and that LAR did not differ from the control group if GDM was treated aiming at tighter glycaemic targets. The GDM1 group (with tighter glycaemic targets) had lower levels of leptin compared to GDM2. We also found a decrease in the level of expression of ANGPTL4 in HUVEC of newborns from women with GDM in comparison with the control group. However, there was no difference in the level of expression of ANGPTL4 between the two groups of GDM with different glycaemic targets.

The most appropriate target levels of glycaemia for the management of GDM are not universally defined [23]. Most organisations [24–26] suggest the targets for glycaemic control for women with GDM based on recommendations from the Fifth International Workshop-Conference on Gestational Diabetes Mellitus [27].

These targets were used by the Maternal-Fetal Medicine Units Network (MFMU) trial showing benefit for the treatment of GDM [28]. We used these targets for group 2.

However, there are no reliable data from controlled trials of lower versus higher target levels of glycaemia to identify ideal glycaemic targets for prevention of fetal risks [29].

The glycaemic targets used in our study for group 1 were tighter in accordance with current Russian guidelines [16]. Our data suggest that achieving tighter glycaemic targets during GDM treatment reduces LAR in the cord blood. However, this data should be interpreted with caution considering the small sample size. The full information about pregnancy outcomes is needed to guide clinical practice regarding target glycaemic levels during pregnancy. Our current study with a small sample size was not designed for this purpose. However, our findings may serve as a confirmation of the cause-and-effect relationship between maternal hyperglycaemia and alteration of LAR in newborns. Our results are in line with the data by Pirc et al. that reported treatment of mild GDM reduces cord blood leptin [12]. The authors hypothesise that hyperleptinaemia of the babies born to women with untreated mild GDM may persist for some time. It could reduce appetite and food intake and might contribute to the phenomenon of catch-down growth seen in macrosomic infants following birth [12, 30].

Follow-up studies are needed to understand the impact of tight glycaemic targets during pregnancy on obesity development in the offspring of women diagnosed with GDM according to IADPSG criteria. It is especially important taking into consideration the evidence that low early-life leptin concentrations may promote faster weight gain in infancy [31–33].

There is controversy about the association of adiponectin levels in cord blood with GDM. Pirc et al. reported decreased levels of adiponectin in the cord blood of newborns from mothers with GDM [12], whereas several other studies, like ours, find no effect of maternal DM on cord blood adiponectin concentration [5, 34, 35]. The reasons for these differences are unclear but may reflect different assay methodologies, different study populations, and different criteria used to diagnose GDM.

The level of expression of LEP in HUVECs turned out to be below the detection threshold. The expression of ADIPOQ was detected in HUVEC samples, but the level of its expression was as low as in negative control samples, which confirms that umbilical vein endothelium is not a place of adiponectin production. We are not aware of any other study addressing the expression of these genes in HUVEC. However, our results are in line with some of the previous studies which have shown that ADIPOQ is not expressed in the placenta [14, 36]. Thus, the levels of cord adiponectin may be attributed to other fetal tissues, while LEP has been shown to be expressed in the placenta [14]. It should be noted that there are also conflicting results of the studies which indicated the expression of ADIPOQ in human placenta [36, 37].

In contrast to LAR changes associated with tight glycaemic targets of treatment of GDM, the level of expression of ANGPTL4 was lower in both the GDM groups regardless of glycaemic targets compared to the control group. Possibly, it is due to the fact that the difference in target glycaemic levels is not significant enough to affect the expression level of ANGPTL4, at least on such a small sample. Another plausible explanation is that the reduced level of activity of ANGPTL4 is transmitted at the genetic level to newborns from their mothers. Maybe, the reduced level of activity of ANGPTL4 contributes to the development of GDM in the mothers, that is, it is the cause, not the consequence of hyperglycaemia.

This hypothesis is supported by the data of Xu et al. on the lower level of ANGPTL4 in patients with type 2 diabetes whose pathogenesis is close to GDM [9]. However, this hypothesis is contradicted by the data of Ortega-Senovilla et al., indicating that maternal serum ANGPTL4 concentrations showed no difference between the control and GDM women [13].

Moreover, opposite to our data, Ortega-Senovilla et al. showed that serum ANGPTL4 concentrations in cord serum were higher in those from GDM than those from control pregnancies [13]. We found no difference in the level of ANGPTL4 in the cord serum. There seems to be no correlation between the levels of ANGPTL4 protein in the cord serum and ANGPTL4 gene expression in one of the fetal tissues (HUVEC). It is plausible that the main source of cord serum ANGPTL4 is some other fetal tissue (e.g., the liver). The functional consequences of the downregulation of ANGPTL4 mRNA levels in HUVECs in GDM remain to be identified.

In addition, other factors besides intrauterine hyperglycaemia may affect the activity of a number of genes, including ANGPTL4, in the fetus. Known factors that affect the weight of the newborn are the body mass index (BMI) of the mother and maternal gestational weight gain. Obviously, these parameters are influenced by the mother's lifestyle (the quantitative and qualitative composition of the diet and the level of physical activity).

It is known that ANGPTL4 can be regulated by diet [38, 39]. The diet interventions leading to NEFA increase in the blood (high-fat diet, a very low-energy diet, and fasting) were shown to upregulate the plasma level of ANGPTL4 [38].

This upregulation has been confirmed likewise in vitro as the expression of ANGPTL4 is upregulated in response to exposure to fatty acids in cell studies [40].

Our study established a significantly lower pregnancy weight gain in GDM patients compared to controls which is obviously due to diet adherence by patients.

Further studies are needed to clarify the cause-and-effect relationship between GDM and the level of expression of ANGPTL4 gene in HUVEC.

The level of C-peptide in the cord blood is commonly used as a marker of fetal hyperinsulinemia [41]. The data presented by HAPO study has shown associations between increasing levels of fasting, 1-hour, and 2-hour plasma glucose obtained on oral glucose-tolerance testing and cord blood serum C-peptide level above the 90th percentile [41]. We did not reveal any difference in the level of C-peptide among the groups. It could be a result of the treatment that was efficient to reduce fetal hyperinsulinemia in both GDM groups or it may be due to a small sample size.

The strength of our study is the design of the RCT of different glycaemic targets for women with GDM which allows at testing cause-and-effect relationships. The weakness of the study, besides its relatively small sample size, is the lack of information about maternal levels of the studied gene expression.

## 5. Conclusion

Our study established positive association of cord leptin levels and LAR with target levels of glycaemia during pregnancy in women with GDM. Further investigation into long-term consequences of cord leptin concentrations is required.

We also found a decrease in the expression of ANGPTL4 in HUVECs of neonates from mothers with GDM. However, we could not prove the causal relationship between intrauterine hyperglycaemia and the expression of the ANGPTL4 gene, given the absence of differences between the level of expression of ANGPTL4 in groups with different glycaemic targets. This relationship remains to be clarified.

## Figures and Tables

**Table 1:** Characteristics of the participants at study entry.

	GDM 1 (*N* = 20)	GDM2 (*N* = 21)	Control (*N* = 25)	*p*	*p* control-GDM1	*p* control-GDM2	*p* GDM1-GDM2
Maternal age, years	30.9 ± 5.4	32.3 ± 5.0	30.8 ± 4.2	0.566			
Prepregnancy BMI, kg/m^2^	25.4 ± 7.2	26.1 ± 6.5	23.4 ± 4.2	0.287			
BP syst, mmHg	120 ± 13	118 ± 12	112 ± 14	0.114			
BP diast, mmHg	76 ± 8	73 ± 10	69 ± 8	0.016	0.003	0.155	0.195
Fasting PG, mmol/L	5.1 ± 0.8	5.0 ± 0.6	4.5 ± 0.4	0.007	0.004	0.003	0.396
OGTT 1 h PG, mmol/L	10.2 ± 1.4	9.9 ± 1.6	6.9 ± 1.9	<0.001	<0.001	<0.001	0.454
OGTT 2 h PG, mmol/L	8.0 ± 1.6	8.8 ± 1.6	5.9 ± 1.5	<0.001	<0.001	<0.001	0.231
Fasting leptin, ng/mL	22.2 ± 20.7	29.5 ± 26.2	26.6 ± 17.0	0.561			
Fasting adiponectin, ng/mL	7.2 ± 3.3	9.1 ± 3.3	8.9 ± 2.5	0.077			

Note: BMI: body mass index; BP: blood pressure; PG: plasma glucose; OGTT: oral glucose tolerance test.

**Table 2:** Blood glucose data from electronic diaries and gestational weight gain.

	GDM 1 (*N* = 20)	GDM2 (*N* = 21)	Control (*N* = 25)	*p*	*p* control-GDM1	*p* control-GDM2	*p* GDM1-GDM2
Gestational weight gain, kg	9.9 ± 4.9	9.5 ± 5.9	15.2 ± 7.8	0.006	0.023	0.023	0.970
BG average, mmol/L^∗^	5.6 ± 0.3	5.9 ± 0.4	6.0 ± 0.5	0.004	0.110	0.893	0.005
Fasting BG, mmol/L^∗^	4.7 ± 0.4	4.8 ± 0.3	4.7 ± 0.3	0.499	0.835	0.421	0.735
1 h postprandial BG, mmol/L^∗^	5.9 ± 0.3	6.4 ± 0.5	6.5 ± 0.7	0.002	0.088	0.818	0.002
Number of BG measurements	140 ± 78	147 ± 60	42 ± 21	0.001	<0.001	<0.001	0.946
% (*N*) treated with insulin	40% (8)	29% (6)	N/A	0.495			

Note: ^∗^derived from electronic diaries filled in by participants (*N* GDM1 = 19, *N* GDM2 = 20, *N* control = 8) during the study period. BG: blood glucose; N/A: nonapplicable.

**Table 3:** Pregnancy outcomes, biochemical markers in the cord blood, and ANGPTL4 gene expression in HUVECs.

	GDM 1 (*N* = 20)	GDM2 (*N* = 21)	Control (*N* = 25)	*p*
Gestational age at delivery, weeks	39.2 ± 1.5	39.3 ± 1.0	39.7 ± 1.0	0.261
Caesarean section, % (*N*)	30% (6)	19% (4)	20% (5)	0.723
Birth weight, g	3572 ± 488	3584 ± 577	3513 ± 555	0.856
Height, cm	52.1 ± 2.5	52.4 ± 2.3	52.1 ± 2.5	0.990
LGA, % (*N*)	20% (4)	23% (5)	12% (3)	0.235
SGA, % (*N*)	5% (1)	9.5% (2)	4% (1)	0.819
Apgar score 1 min	7.5 ± 0.7	7.7 ± 1.1	7.7 ± 0.6	0.204
Apgar score 5 min	8.6 ± 0.5	8.7 ± 0.9	8.8 ± 0.4	0.208
Glucose, mmol/L	4.7 ± 1.2	5.3 ± 1.3	4.5 ± 1.2	0.203
C-peptide, ng/mL	0.8 ± 0.5	1.0 ± 0.6	0.9 ± 0.4	0.379
Leptin, ng/mL	8.8 ± 6.6^a^	18.3 ± 16.1	10.6 ± 10.4	0.042
Adiponectin, ng/mL	15.9 ± 11.5	16.3 ± 14.4	18.3 ± 14.3	0.843
LAR	0.97 ± 1.31	1.70 ± 1.66^b^	0.72 ± 0.46	0.038
ANGPTL4 in cord serum, ng/mL	19.9 ± 15.0	14.1 ± 4.5	13.9 ± 5.2	0.248
ANGPTL4 relative expression in HUVECs	23.1 ± 25.6^c^	21.5 ± 25.8^c^	98.3 ± 104.6	0.001

Notes: LAR: leptin/adiponectin ratio; LGA: large for gestational age; SGA: small for gestational age. LGA was defined by a birth weight exceeding the 90th percentile on standard charts. SGA was defined by a birth weight below the 10th percentile on standard charts. ^a^*p* < 0.05 versus GDM2; ^b^*p* < 0.05 versus the control group; ^c^*p* < 0.01 versus the control group.

## Data Availability

The data used to support the findings of this study are available from the corresponding author upon request.
